# Feature Extraction of 3T3 Fibroblast Microtubule Based on Discrete Wavelet Transform and Lucy–Richardson Deconvolution Methods

**DOI:** 10.3390/mi13060824

**Published:** 2022-05-25

**Authors:** Haoxin Bai, Bingchen Che, Tianyun Zhao, Wei Zhao, Kaige Wang, Ce Zhang, Jintao Bai

**Affiliations:** 1 State Key Laboratory of Photon-Technology in Western China Energy, International Collaborative Center on Photoelectric Technology and Nano Functional Materials, Institute of Photonics & Photon Technology, Northwest University, Xi’an 710127, China; 202032221@stumail.nwu.edu.cn (H.B.); bingchenc03@gmail.com (B.C.); wangkg@nwu.edu.cn (K.W.); baijt@nwu.edu.cn (J.B.); 2 School of Automation, Northwestern Polytechnical University, Xi’an 710129, China; zhaoty@nwpu.edu.cn

**Keywords:** DWDC method, super-resolution, 3T3 fibroblasts microtubule, PSNR, SSIM

## Abstract

Accompanied by the increasing requirements of the probing micro/nanoscopic structures of biological samples, various image-processing algorithms have been developed for visualization or to facilitate data analysis. However, it remains challenging to enhance both the signal-to-noise ratio and image resolution using a single algorithm. In this investigation, we propose a composite image processing method by combining discrete wavelet transform (DWT) and the Lucy–Richardson (LR) deconvolution method, termed the DWDC method. Our results demonstrate that the signal-to-noise ratio and resolution of live cells’ microtubule networks are considerably improved, allowing the recognition of features as small as 120 nm. The method shows robustness in processing the high-noise images of filament-like biological structures, e.g., the cytoskeleton networks captured by fluorescent microscopes.

## 1. Introduction

### 1.1. Research Background

In recent years, as key components, microdevices have been widely used in the development of biological and biomedical techniques for manipulating, mixing, separating, and sensing biological targets. At the same time, increasingly stringent requirements have been placed on imaging techniques involving high signal-to-noise ratio and structural resolution. Currently, the most commonly used devices for the high-resolution imaging of biological or biomedical targets include confocal microscopes [1], stimulated emission depletion (STED) microscopes [2], and structured light illumination microscopes (SIM) [3] etc. Furthermore, many algorithms have been developed to improve the spatial resolution and signal-to-noise ratio (SNR) of biological images, including degenerate-model-based algorithms (e.g., deconvolution [4,5,6,7,8]), mathematical transformation-based algorithms (e.g., spectrum analysis [9,10], DWT analysis [11,12,13,14,15,16]), and machine-learning-based algorithms (e.g., deep learning [17,18,19]). However, most of these algorithms focus on a single task, e.g., inhibiting noise, identifying structure contours, or improving resolution. Furthermore, these methods normally require the target images to be clear, with relatively low levels of noise.

Nevertheless, many fundamental and representative biological structures are small and irregular and suffer from significant noise backgrounds during imaging. For example, the microtubule of the fibroblast is a fundamental cell structure and plays an important role in cellular responses to external stimuli. It has filament-like structures with a width of ~25 nm [20] and forms a densely packed network in nature [21]. These factors make it difficult to distinguish a single microtubule filament and track its dynamics during various biological processes, especially against noisy backgrounds, in which fluorescence signals due to emitted background light and autofluorescence originate from the areas above and below the focal plane can decrease the SNR of image. For isotropic or quasi-isotropic features, e.g., round-shaped and nanometer-sized exosomes, a deconvolution-based algorithm can effectively improve the structural resolution [22,23]. For densely packed networks (e.g., the microtubule), researchers are still paying more attention on high-performance image-processing methods [24], to achieve higher resolution and SNR.

### 1.2. Previous Works

Deconvolution methods, including the Lucy–Richardson (LR) algorithm [25,26,27,28], the fast thresholded Landweber (FTL) algorithm, the generalized expectation maximization (GEM) algorithm, etc., are commonly used in improving the image resolution and quality. The LR method was widely used in image processing accompanied by STED microscopy. For instance, in 2006, Willig et al. studied green fluorescent protein (GFP)-labeled viruses with STED microscopy and the LR method, achieving a lateral resolution of about 70 nm [29]. In 2007, Hell used the LR method to increase the spatial resolution of STED microscopy to 20–30 nm [30]. The GEM algorithm was advanced in 2006 by Bioucas-Dias et al. to process macroscopic images [31]. Although the authors found that the GEM method could improve image quality, the algorithm only compares the SNR before and after processing, which cannot ensure the original image intensity distribution before and after image processing. The FTL algorithm is a fast variational deconvolution algorithm that minimizes a quadratic data term. Vonesch et al. used FTL to process confocal images of a neuron cell [32]. They found that the FTL algorithm could achieve an eight-decibel improvement in ten iterations, with an insignificant increase in the image SNR. However, deconvolution methods by themselves may lead to over-processing and spurious images, especially in images with poor SNR.

By contrast, the wavelet method has been commonly applied for denoising (e.g., the expectation maximization (EM) algorithm [33,34]) and the extraction of featured structures through scales [35]. For instance, the EM algorithm utilizes both wavelet transform and fast Fourier-transform to improve the SNR of images. It can increase the SNR of a macroscopic image from 3 dB to ~7 dB after 8 to 10 iterations.

## 2. Methodological Principles and Process

### 2.1. Target of Image Process

In this investigation, we developed a new algorithm named as DWDC method, which combines DWT and Lucy–Richardson deconvolution. With this method, the spatial resolution of a typical biological image (high noise, blurred, and unclear) can be increased with improved SNR, and the features of filament-like structures can be extracted.

**Figure 1:**
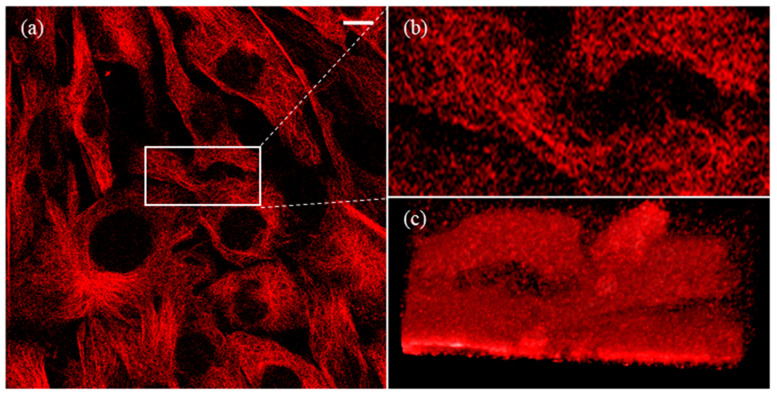
Original confocal image of 3T3 cell microtubule. (**a**) Image has 512 × 512 pixels, with a dot pitch of 250 nm. (**b**) The zoomed-in image of the white box. (**c**) The three-dimensional (3D) reconstruction of (**b**). The white scale bar represents 10 µm (for further details on the 3D structure of Figure 1 (**c**), see Supplementary video S1).

Figure 1a shows a confocal fluorescence image of 3T3 fibroblast microtubule networks, which were taken using Nikon A1 microscope and Olympus 100X oil immersion lens (NA 1.4) (Nikon Corporation, Tokyo, Japan). The excitation light wavelength is 640 nm, and the emission peak is around 674 nm for the SiR-Tubulin dye (Cytoskeleton, Inc. Denver, CO, USA). Each fluorescence image has 512 × 512 pixels, with a dot pitch of 0.25 µm. The fluorescence image’s bits per pixel (BPP) is 16 and the contrast ratio (CR) is 6000:1. For 3D reconstruction, twenty images were captured by z-stacking, with a 1-micrometer vertical interval. It is obvious that the branch of microtubule structures is highly contaminated by noise and the structures are clearly bold (Figure 1b,c). We can hardly distinguish the topological structures due to the fragmental distribution of fluorescent intensity. Structural features reflecting cell–cell interactions are indistinguishable from the figures. Since the featured scale of biological samples is comparable to or even smaller than the image pixel pitch (250 nm), the actual distribution of biological structures can be affected by the noise distribution. It is necessary to remove as much noise as possible before analyzing biological activities.

### 2.2. Methods and Process

An optical image is a convolution of an object with the point spread function (PSF) of an optical system [36]. If M is the matrix of the image,

(1) M=P⊗S+N

where P is the PSF of the optical system, S is the light distribution according to the object and N is the measurement noise of the optical system. If the size of the PSF is larger than the size of the mesostructure of the actual object, the imaging result has an insufficient spatial resolution to reveal the detail of the original object. Accordingly, the image after the optical system is blurred relative to the actual object.

**Figure 2:**
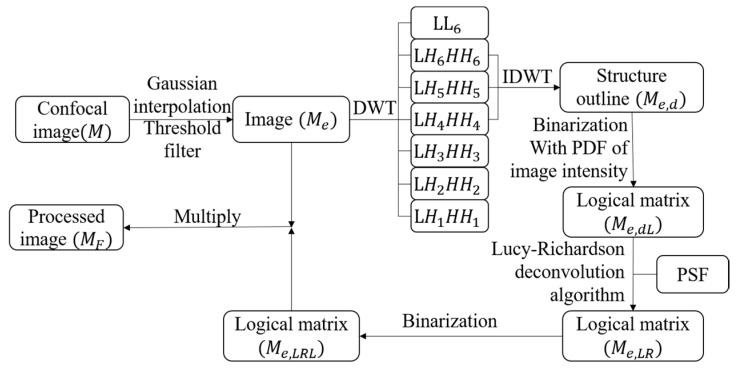
Schematic of DWDC method.

The DWDC method advanced in this investigation utilizes both LR and DWT, as presented in Figure 2. Firstly, the image was processed using Gaussian interpolation and threshold analysis. DWT was then applied to suppress noise level and extract characteristic microtubule structures on the basis of scale analysis. In DWT wavelet processing, $LL_{n}$ is the approximate wavelet decomposition term, $LH_{n}$ is the detailed wavelet decomposition terms in the x-direction and y-direction, and $HH_{n}$ is the detailed wavelet decomposition terms in the diagonal direction. The subscripts of the terms represent the order of wavelet decomposition. During inverse DWT, only four–six order terms were included in this investigation. Consequently, the outline of the representative structures was distinguished by binarization with threshold processing, i.e., logical matrix 1 ($M_{e,dL}$). Application of the deconvolution method shrank the outline and further enhanced the spatial resolution, i.e., logical matrix 3 ($M_{e,LRL}$). The image was then processed with repeated binarization, threshold analysis, and Gaussian interpolation before finalization. The overall processed image $M_{F}$ can be obtained by $M_{e}\cdot M_{e,LRL}$.

#### 2.2.1. Expansion of Image by Gaussian Interpolation

To increase the resolution of the image, we first reduced the dot pitch. 3D Gaussian interpolation [37,38] was applied to expand the size of the image with reduced pitch. The original 20 images of 512 × 512 pixels were expanded twice to 80 expansion images ($M_{e}$) of 2048 × 2048 pixels. The Gaussian function is:

(2) $$g=g_{0}\exp\left[-\frac{{\left(x-x_{c}\right)}^{2}+{\left(y-y_{c}\right)}^{2}}{2r_{⊥}^{2}}-\frac{{\left(z-z_{c}\right)}^{2}}{2r_{∥}^{2}}\right]$$

where $r_{⊥}=\left(0.61\lambda_{e}\right)⁄\;N_{A}$ and $r_{∥}=\left(4n\lambda_{e}\right)⁄\left(2N_{A}{}^{2}\right)$ are the lateral and axial Gaussian radius, respectively [39,40], $N_{A}$ is numerical aperture of lens, and $\lambda_{e}$ is the wavelength of the excitation beam. After the expansion, the dot pitch was reduced to 63 nm.

#### 2.2.2. Extraction of Microtubule Structures by DWT

**Figure 3:**
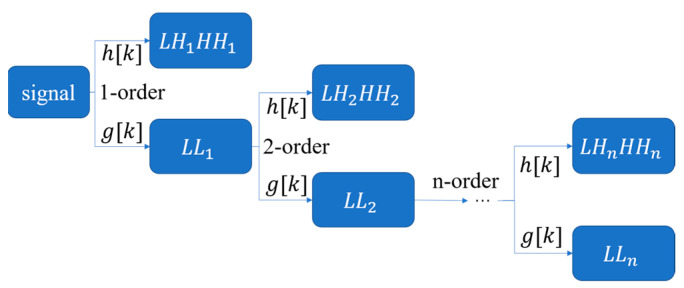
Wavelet decomposition process, where g[k] is a low-pass filter, and h[k] is a high-pass filter.

In this investigation, we applied DWT to extract microtubule structures [41] from the expanded image matrix. Relative to the Fourier-transform-based image analysis, which can only filter images globally, DWT can extract different image structures based on their local scale and intensity distributions. This is more suitable for processing cellular microtubule structures, which are highly local and anisotropic. When performing wavelet decomposition, the information corresponding to the scale function is usually filtered by a low-pass filter, and the information corresponding to the wavelet function is filtered by a high-pass filter, as shown in Figure 3. At the same time, the scale information obtained by the low-pass filter can be used as the generating function of the wavelet function and the scale function of the next stage. The information corresponding to the scale function represents the low-frequency component in the original signal, which represents the coarse information of the original signal; the information corresponding to the wavelet function represents the high-frequency component in the original signal, which represents the detailed information component of the original signal.

**Figure 4:**
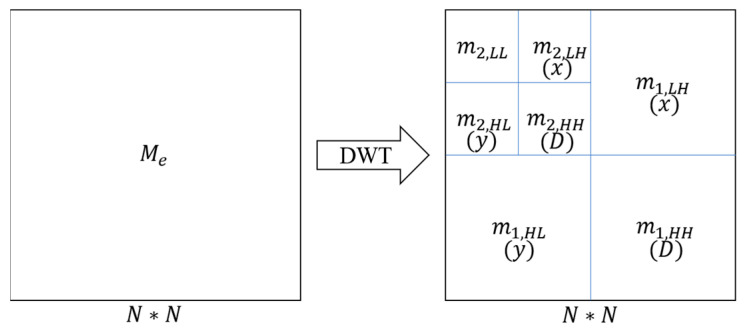
Diagram of second-order wavelet decomposition on $M_{e}$. The left side is the image matrix for decomposition, and the right side shows the decomposed matrices. N is the size of the image matrix. N= 2048 in this investigation.

For a two-dimensional image matrix, wavelet decomposition is processed in three directions, i.e., horizontal, vertical, and diagonal directions, as:

(3) $$\begin{array}{ll} M_{e}\left(x,y\right)= & c_{n}m_{n,LL}\left(x,y\right)+d_{n,x}m_{n,LH}\left(x,y\right)+d_{n,y}m_{n,HL}\left(x,y\right)\\ & +d_{n,D}m_{n,HH}\left(x,y\right)+\cdots +d_{2,x}m_{2,LH}\left(x,y\right)+d_{2,y}m_{2,HL}\left(x,y\right)\\ & +d_{2,D}m_{2,HH}\left(x,y\right)+d_{1,x}m_{1,LH}\left(x,y\right)+d_{1,y}m_{1,HL}\left(x,y\right)\\ & +d_{1,D}m_{1,LH}\left(x,y\right) \end{array}$$

where $c_{n}$ is the approximate wavelet coefficient after *n*th-order discrete wavelet decomposition, and $d_{n,x}$, $d_{n,y}$, and $d_{n,D}$ are the detailed wavelet coefficients of level n in horizontal, vertical, and diagonal directions, respectively, as shown in Figure 4. $m_{n,LL}$ is the approximate information. $m_{n,LH}$ is the detailed information in the x-direction, $m_{n,HL}$ is the detailed information in the y-direction, and $m_{n,HH}$ is the detailed information in the diagonal direction.

The values are obtained from the image $M_{e}$ by the *n*-order DWT. $m_{n,LL}$, $m_{n,LH}$, $m_{n,HL}$, and $m_{n,HH}$ have the following formats:

$$m_{n,LL}\left(x,y\right)=\sum_{k}m_{n-1,LL}\left(2x-k,2y-k\right)g\left[k\right]g\left[k\right];k\in Z$$

$$m_{n,LH}\left(x,y\right)=\sum_{k}m_{n-1,LL}\left(2x-k,2y-k\right)g\left[k\right]h\left[k\right];k\in Z$$

$$m_{n,HL}\left(x,y\right)=\sum_{k}m_{n-1,LL}\left(2x-k,2y-k\right)h\left[k\right]g\left[k\right];k\in Z$$

$$m_{n,HH}\left(x,y\right)=\sum_{k}m_{n-1,LL}\left(2x-k,2y-k\right)h\left[k\right]h\left[k\right];k\in Z$$

where $m_{0,LL}\left(x,y\right)=M_{e}\left(x,y\right)$. After DWT, Equation (1) can be expressed as:

(4) $$M_{e,d}={\left(P⊗S\right)}_{d}+N_{d}$$

The image information can be further divided into two parts in scale space, i.e., the scales related to the desired structures (denoted as $M_{e,d}\left(x,y\right)$) and the scales related to undesired structures (denoted as $M_{e,ud}\left(x,y\right)$), which can also be considered as noise structures. Thus,

(5) $$M_{e}\left(x,y\right)=M_{e,d}\left(x,y\right)+M_{e,ud}\left(x,y\right)$$

where

(6) $$M_{e,d}\left(x,y\right)=\sum_{n=n_{l}}^{n=n_{h}}\left[d_{n,x}m_{n,LH}\left(x,y\right)+d_{n,y}m_{n,HL}\left(x,y\right)+d_{n,D}m_{n,HH}\left(x,y\right)\right]$$

where $n_{l}$ and $n_{h}$ correspond to the lower and higher bounds of DWT orders of the desired structures. For instance, if the desired structures have a characteristic size between 16 and 64 pixels, we have $n_{l}=4$ and $n_{h}=6$. According to DWT decomposition, the image structures within desired scale ranges (or frequency ranges) can be retained in each local position. The undesired image structures, e.g., noise (which normally has high-frequency components) and image distortions due to nonuniform illumination (which has low-frequency components), can be removed from the images without the requirement of knowing their detailed distribution.

#### 2.2.3. Binarization of Image

When we obtained the desired structures extracted by DWT, logic processing was carried out. The threshold value (χ) was selected according to the probability density distribution of image intensity. The binarization image was thus obtained as a logical matrix:

(7) $$M_{e,dL}\left(i,j\right)=\{\begin{array}{c} 0\;(M_{d}\left(i,j\right)<\chi )\\ 1\;\left(M_{d}\left(i,j\right)\geq \chi \right) \end{array}$$

After the processing, the image noise was further inhibited and the outline of demanded structure was highlighted.

#### 2.2.4. Resolution Improvement by Lucy–Richardson (LR) Deconvolution Method

The logic matrix obtained in this way was not detailed enough and the structural resolution was not, apparently, improved. We then used the Lucy–Richardson deconvolution method to further process the image. Instead of directly applying LR on the image after DWT analysis, in this investigation, we applied LR on the logic matrix ($M_{e,dL}$) to restore the sketch of the filament structures. This approach can prevent the spread of high-intensity structures that affect the low-intensity structures and lead to spurious images or overprocessing.

LR method is developed on the basis of Bayesian theory [42], Poisson distribution, and maximum likelihood estimation. The overall expression formula of LR deconvolution method algorithm is as follows:

(8) $$M_{mid}\left(x,y\right)=M_{k}\left(x,y\right)\cdot \left\{\left[\frac{M_{e,dL}\left(x,y\right)}{M_{k}\left(x,y\right)⊗P\left(x,y\right)}\right]⊗P\left(-x,-y\right)\right\}$$

where $M_{k}\left(x,y\right)$ is the kth iteration of logic matrix $M_{e,dL}$ and $M_{mid}\left(x,y\right)$ are intermediate results. In each iteration of optimization, a scale factor f is applied to evaluate the effect of this processing, according to the image difference before and after the iteration, as:

(9) $$f=\frac{\left|M_{mid}\left(x,y\right)-M_{k}\left(x,y\right)\right|\times \left|M_{k}\left(x,y\right)-M_{k-1}\left(x,y\right)\right|}{\left|M_{k}\left(x,y\right)-M_{k-1}\left(x,y\right)\right|\times \left|M_{k}\left(x,y\right)-M_{k-1}\left(x,y\right)\right|}$$

Thus, the kth iteration of the image during LR deconvolution can be obtained as:

(10) $$M_{kmax}\left(x,y\right)=M_{mid}\left(x,y\right)+f*\left[M_{mid}\left(x,y\right)-M_{kmax-1}\left(x,y\right)\right]$$

where kmax is the maximum number of iterations.

During the deconvolution, one of the most important prerequisites is the evaluation of the actual PSF of the optical system. During LR deconvolution on the image, the PSF of the confocal imaging system is a two-dimensional (2D) Gaussian function, which can be expressed as $P=P_{0}\exp\left[-[{\left(x-x_{c}\right)}^{2}+{\left(y-y_{c}\right)}^{2}/2{\left(\Delta r\right)}^{2}\right]$, where $\Delta r=\xi r_{⊥}$ is the actual PSF for LR deconvolution and ξ is an experience coefficient that should be determined by a numerical experiment. However, since the DWT is followed by a binarization to extract the structure contour, the PSF of the image is broadened with a larger width than that of the original system. Furthermore, in the iterative process of deconvolution, background noise can also be gradually amplified, resulting in spurious images or overprocessing. Therefore, optimizing the number of iterations (i.e., kmax) and threshold deviations (damping coefficient) is important in the application of LR deconvolution. The evaluation of the image after deconvolution is highly arbitrary, depending on the desired structures of the image. In many cases, the restoration quality of an image cannot be simply evaluated by peak signal-to-noise ratio (PSNR) or structural similarity index measure (SSIM). Therefore, in this investigation, the deconvolution parameters were also determined by numerical experiments.

#### 2.2.5. Post-Processing

After LR algorithm, the processed logic matrix showed nonlinear characteristics. Thus, we carried out another binarization process on $M_{kmax}$, with the threshold value χ=1. The secondary logic matrix can be obtained as:

(11) $$M_{e,LRL}\left(x,y\right)=\{\begin{array}{c} 0\;\;\;(M_{kmax}\left(x,y\right)<\chi )\\ 1\;\;\;\left(M_{kmax}\left(x,y\right)\geq \chi \right) \end{array}$$

Finally, the final result ($M_{F}\left(x,y\right)$) of DWDC processing can be obtained by multiplying the expanded image $M_{e}$ by $M_{e,LRL}$, to extract the microtubule structure of the 3T3 cell, i.e.,

(12) $$M_{F}\left(x,y\right)=M_{e}\left(x,y\right)*M_{e,LRL}\left(x,y\right)$$

## 3. Experimental Results

### 3.1. Ground Truth Verification

**Figure 5:**
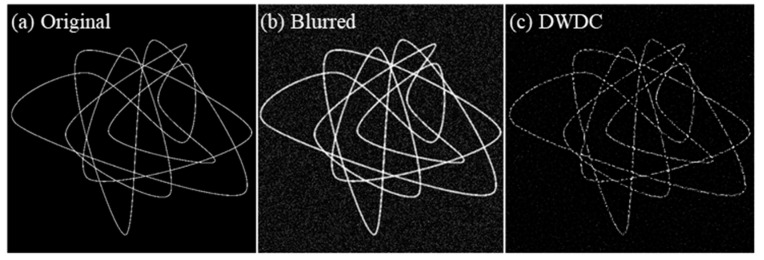
Comparison between the (**a**) ground truth image, (**b**) the blurred and noised image, and (**c**) the DWDC-processed image. Here, Gaussian noise with an average value of 0 and a standard deviation of 2 was applied to the ground truth to generate (**b**).

The DWDC method algorithm was first tested by ground truth images, which are artificially generated bundles of filament-like structures, as shown in Figure 5a. The ground truth structures had a typical width of 63 nm. To mimic the actual biological image captured by a confocal microscope, a Gaussian blur and a Gaussian noise background were applied to the ground truth image, as shown in Figure 5b. The radius of the Gaussian blur function was 270 nm. The Gaussian noise had an average value of 0 and a standard deviation (STD) of 2. Before the DWDC processing, the blurred and noised image had a PSNR of 19.5773 dB and an SSIM of 0.0887 (see Table 1 for details). The latter was especially poor. After the DWDC processing, both the PSNR and SSIM increased to 20.6765 dB and 0.7082, respectively. The processed image in Figure 5c reproduces the skeleton of the ground truth with high visual consistency.

**Figure 6:**
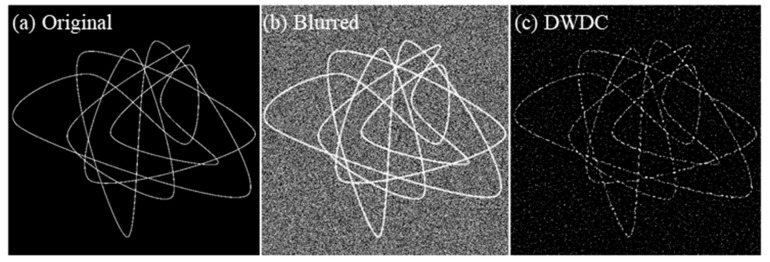
Comparison between the (**a**) ground truth image, (**b**) the blurred and noised image, and (**c**) the DWDC processed image. Here, Gaussian noise with an average value of 40 and a standard deviation of 10 was applied to the ground truth to generate (**b**).

**Figure 7:**
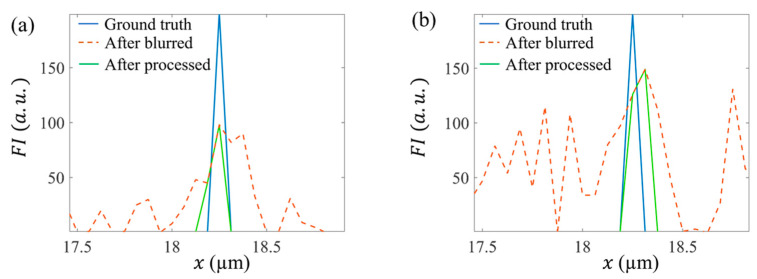
Comparison between the profiles in the ground truth image, blurred and noised image, and DWDC processed images; (**a**) corresponds to Figure 5, and (**b**) corresponds to Figure 6.

We further increased the noise level by applying a Gaussian noise with an average value of 40 and an STD of 10, to test whether a highly noised image could still be improved by the DWDC method. The results are presented in Figure 6. The strong noise (Figure 6b) broke the structure of the ground truth (Figure 6a) and led to misleading distributions, e.g., larger width and more peaks, as can be clearly observed from the profiles in Figure 7b. The PSNR and SSIM of the blurred and noised image are only 12.1049 dB and 0.0297. However, after the DWDC processing, both the PSNR and the SSIM increased to 17.8139 dB and 0.3251, respectively. The processed image in Figure 6c also reproduces the skeleton of the ground truth with acceptable visual consistency. The improvement is very clear.

Figure 7 shows the profiles of the image intensity along a horizontal line under both the low noise (Figure 5) and high noise (Figure 6). In both cases, the profiles after DWDC processing showed significantly smaller FWHM than the blurred and noised profiles. In the low-noise case, the FWHM in the processed profile was 89.1 nm, which was close to that of the ground truth. In the high-noise case, the FWHM in the processed profile was 119.3 nm, which also approached that of the ground truth and exhibited clear resolution improvement relative to the blurred and noised image. It should be noted that although most of the noise peaks were removed, it is too difficult to remove all the noise in a highly noisy image, and some peaks may remain.

In Table 1, we list four pairs of comparisons before and after DWDC processing, with the different noise levels. In these cases, the PSNR improved obviously with greater noise and SSIM improved by a factor of nearly 10.

### 3.2. Expansion of Image by Gaussian Interpolation

**Figure 8:**
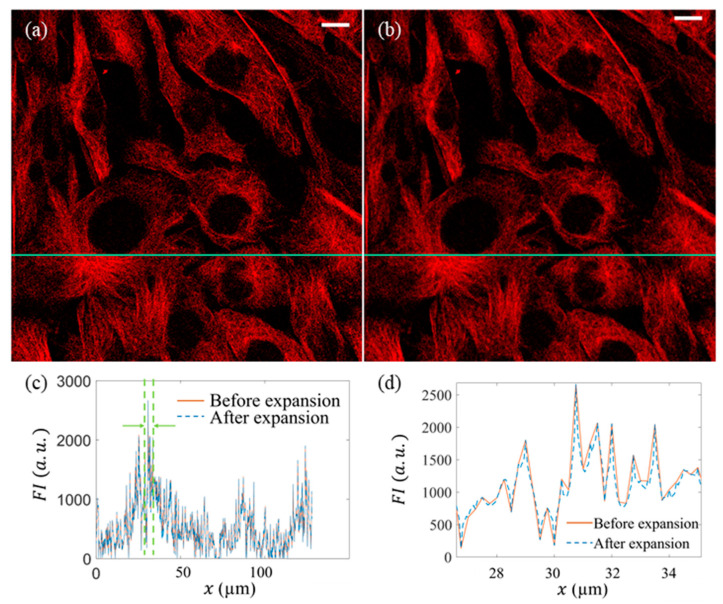
Comparison between the original and expanded images. White scale bars represent 10 µm. (**a**) Original image; (**b**) expanded image; (**c**) distributions of fluorescent intensity (NFI) on the corresponding horizontal and vertical positions before and after expansion; (**d**) zoom-in of (**c**).

A direct comparison between the original 512 × 512 image (i.e., M matrix) and the expanded 2048 × 2048 image ($M_{e}$) is presented in Figure 8. First, visually, the expanded image shows the same high consistency as the original image, as shown in Figure 8a,b. At the corresponding positions, the intensity distributions along the selected row and column both show high similarity (Figure 8c,d), which shows that the details of the original image were reserved.

### 3.3. Extraction of Microtubule Structures by DWT

In this investigation, we used Coiflet 3 wavelet function to decompose the image up to the sixth order, where 3 represents the subtype of the Coiflet wavelet. Because the microtubule structure in the image had a width of 20 to 60 pixels, in the extraction, we only kept 4–6 order components, i.e.,

(13) $$M_{e,d}\left(x,y\right)=\sum_{n=4}^{n=6}[d_{n,x}m_{n,LH}\left(x,y\right)+d_{n,y}m_{n,HL}\left(x,y\right)+d_{n,D}m_{n,HH}\left(x,y\right)]$$

**Figure 9:**
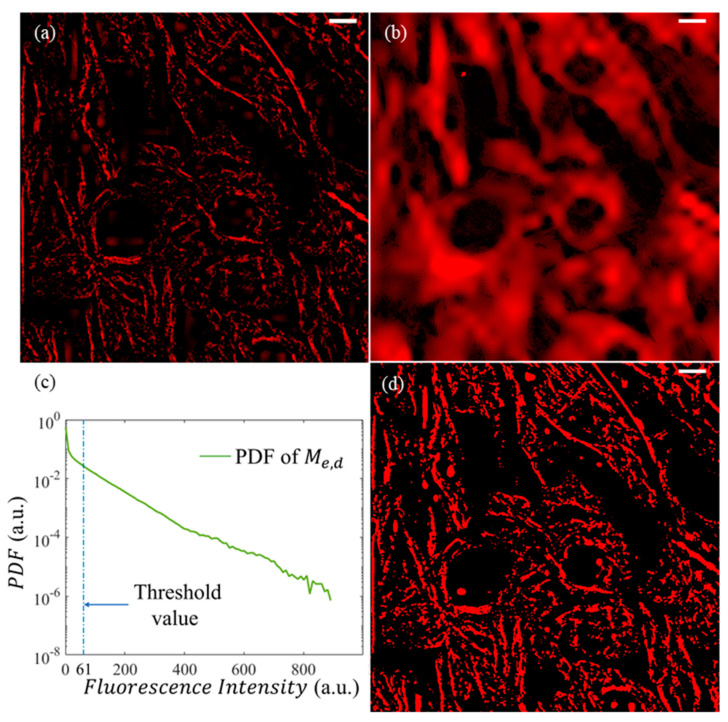
Extraction of microtubule structures by DWT. (**a**) Extracted components after DWT analysis. (**b**) Undesired components that should be abandoned. (**c**) Probability density function (PDF) of $M_{e,d}$. (**d**) Logical matrix ($M_{e,dL}$ ) of the structure after DWT analysis. The white scale bars represent 10 µm.

In contrast to the expanded image (Figure 8b), the reconstructed image shown in Figure 9a clearly reserves the filament-like microtubule structure. The undesired components (Figure 9b), which make the image blurry and noisy, were successfully removed. After DWT analysis, a binarization process was applied, according to the probability distribution of $M_{e,d}$, to extract the sketch of the desired structures. The probability distribution of $M_{e,d}$ is plotted in Figure 9c. Following the numerical experiments, we only retained the top 15% of the image intensity. Thus, the corresponding threshold value related to Figure 9c was estimated to be χ=61; the sketch of the microtubule can be clearly observed in Figure 9d.

### 3.4. Resolution Improvement by LR Deconvolution

**Figure 10:**
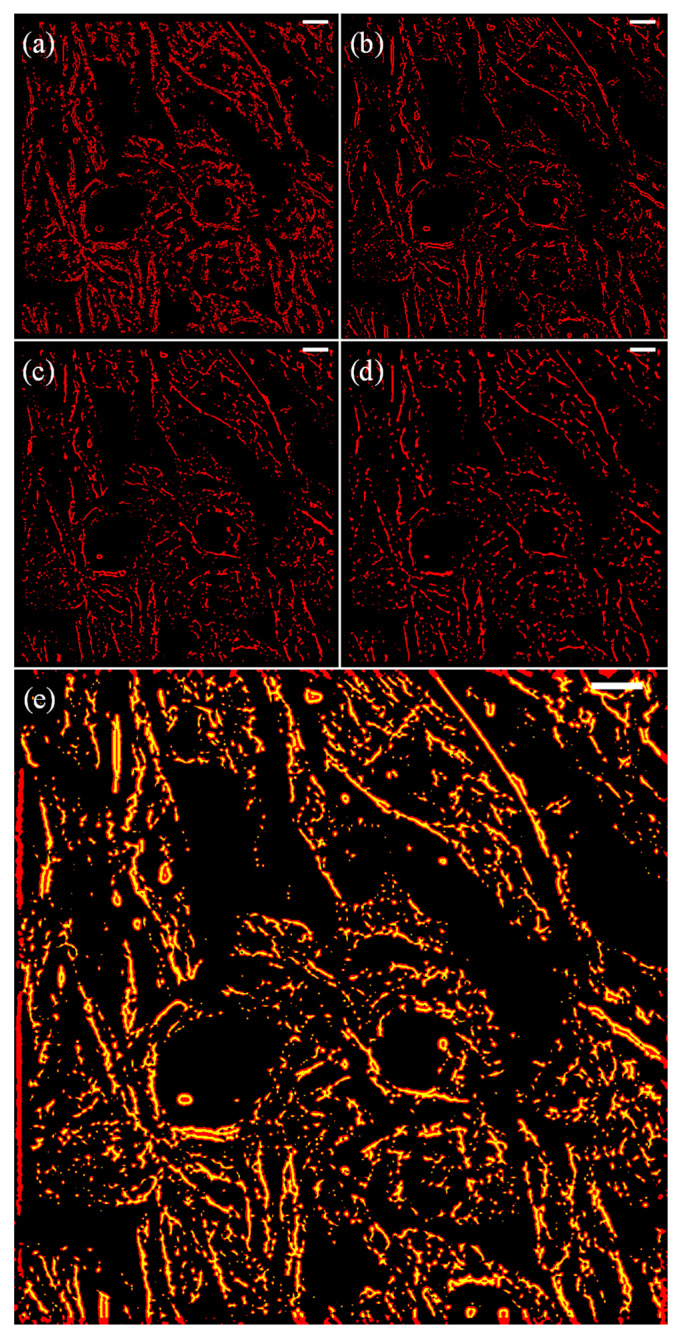
Different empirical coefficients corresponding to the deconvolution results. White scale bars represent 10 µm. (**a**) ξ=0.5; (**b**) ξ=2.5; (**c**) ξ=4.5; (**d**) ξ=6. (**e**) The comparison between pre-processing and post-processing of the logic matrix used to extract the microtubule structure in the original image. Here, ξ=2.5 is applied. The white scale bars represent 10 µm.

At this stage, the sketch of the structure was still wide, and the spatial resolution of the structures was below our expectations (Figure 9d). Subsequently, we used the LR deconvolution algorithm to further process the logical matrix. During the numerical experiments, we used different ξ, maximum iteration kmax, and damping coefficient to optimize the outcome (i.e., narrow and consistent structural features). The representative results are listed in Figure 10a–d. Notably, all the images in Figure 10 were binarized after LR deconvolution.

When ξ=0.5, the applied Gaussian radium for deconvolution Δr was only half of the theoretical value $r_{⊥}$. The continuous structures of the logical matrix became fragmented and hollow (Figure 10a). When ξ was increased to 2.5, significant shrinkage of the logical matrix structures was achieved with at the expense of continuous network structures (Figure 10b). The optimal outcome was obtained when kmax and the damping coefficient were 10 and 0.01, respectively (Figure 10e). It should be noted that before applying the DWDC method, the optimal ξ must be calculated, since a mismatching ξ can lead to invalid or even over-processed images. For different optical systems, the PSF and the optimal ξ are different. For a different optical system, it may take a few hours to locate the optimal ξ.

### 3.5. Image after Processing

**Figure 11:**
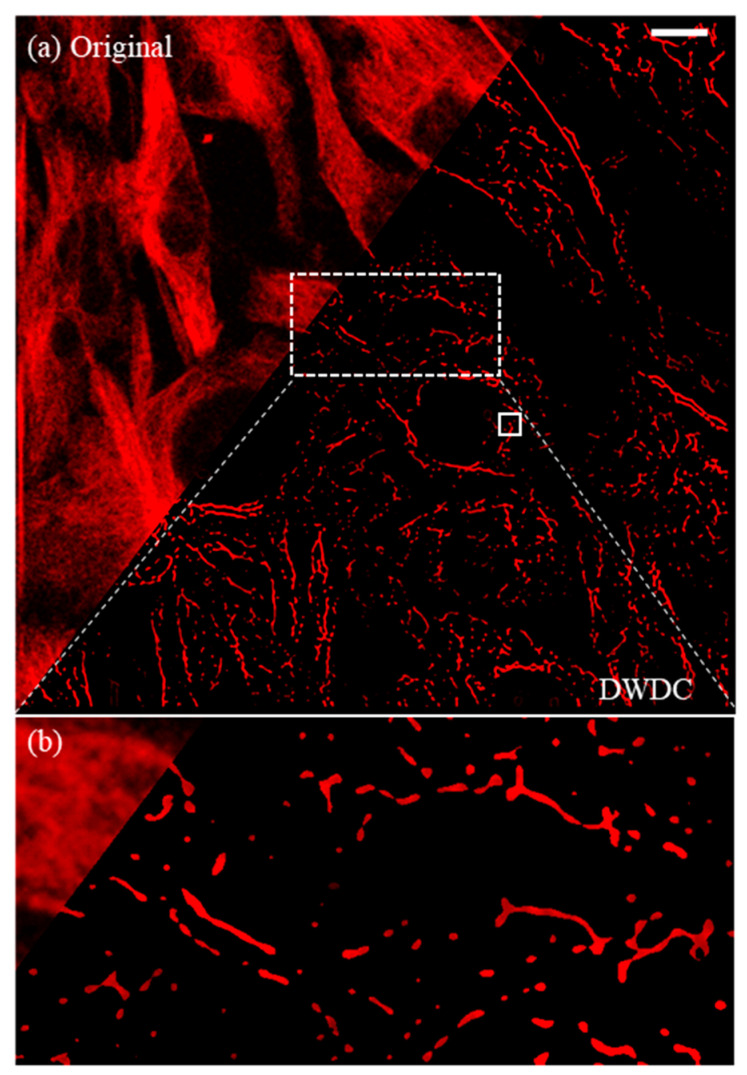
Comparison of original image and image processed after DWDC method. (**a**) Direct comparison before and after the DWDC method. The white scale bar represents 10 µm. (**b**) Local processing results of the DWDC method in the box of the dashed line of (**a**) for comparison with Figure 1b (see more details for 3D structure of Figure 11b in Supplementary video S2).

By extracting the structure of the logical matrix, the microtubule structure of 3T3 cells was obtained (Equation (12) and Figure 11a). The final image rendered the clear filament-like mesh structures of the microtubule in the 3T3 cells, with good contrast and low noise, as shown in Figure 11b. A clearer comparison between the original images and the processed images can be found in the 3D microtubule structures of the 3T3 cells in the Supplementary videos.

**Figure 12:**
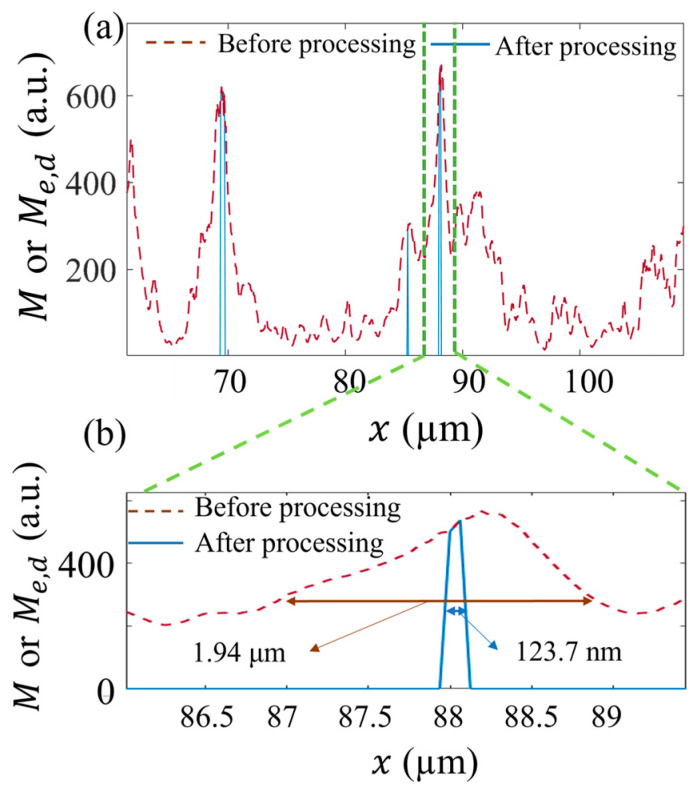
Comparison of FWHM between original image and image processing after DWDC method in the box of the solid line in Figure 11 (**a**). M and $M_{e,d}$ are the image intensities of the 3T3 cell image; (**b**) is the zoom-in of (**a**).

In Figure 12, we compare the distributions of the fluorescence intensity at the same positions of the original and processed images, which show a fifteen-fold improvement in spatial resolution, from 1.94 μm to 123.7 nm, as evaluated by the full width at half maximum (FWHM) of the structure. The image after DWDC processing achieved a super-resolution level, i.e., beyond the optical diffraction limit.

Notably, the PSNR, a commonly used criterion for evaluating the noise level of images before and after processing, was −48.8. The SSIM, which is another widely used parameter, was only 0.0155. This implies that PSNR and SSIM, as the common judging standards, may not be the gold standard with which to evaluate feature extraction algorithms in biomedical and biological applications if drawing a comparison between original and processed images directly.

**Figure 13:**
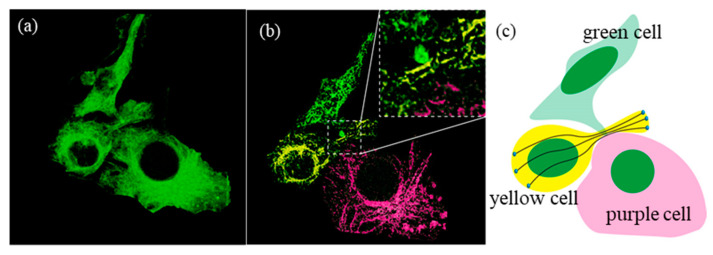
Schematic of cell collision and crossing through the 3D reconstruction of the images. (**a**) 3D reconstruction from the original images, (**b**) 3D reconstruction from the processed images, and (**c**) schematic of the relationship between the three cells.

The difference before and after the DWDC processing from the 3D reconstruction can be seen more clearly. In Figure 13a, which is reconstructed from the original images, only the surfaces of the cells can be distinguished, not the microtubule structures. After processing, the previously unclear images of the microtubule network structure (Figure 1b,c) became considerably more distinguishable (Figure 13b), and more biological information was revealed. As an example, our results demonstrate that two cells (i.e., the green- and purple-colored cells) formed a cell–cell connection. When the third cell (yellow) passed through the gap between these two cells (Figure 13c), this remodeled its microtubule network and indicated that mechanical force was induced by the cell–cell collision [43]. Since it is widely accepted that propagating mechanical cues during the collective movement of population cells can activate mechano-signaling and regulate cellular behavior [44], in which the remodeling of cytoskeleton networks plays important roles, our approach shows potential for deciphering dynamic cytoskeleton network reorganization and remodeling at the single-molecule level, even with conventional high-resolution imaging techniques.

## 4. Conclusions

In this investigation, we introduce the use of the DWDC method, which was developed based on the discrete wavelet transform and Lucy–Richardson algorithm, to extract the microtubule structures of 3T3 cells from confocal images. Using the DWDC method, a sequence of image processing steps was applied, including Gaussian interpolation, discrete wavelet transform, Lucy–Richardson deconvolution, binarization, and probability density analysis. The skeletons of the filament-like microtubule structures were distinguished with significantly reduced width. Finally, the microtubule structures can be extracted with much higher spatial resolution. The microtubule structure in the original image, which had a FWHM of up to 1.94 µm, was reduced to 123.7 nm after processing with the DWDC method. The improvement in the structural resolution was around fifteen-fold. In the numerical experiments, the PSNR of the original images was enhanced by up to 5.7 dB and the SSIM was improved by a factor of 10. Compared with the single use of discrete wavelet transform or Lucy–Richardson algorithm for image processing, the composite image processing method can effectively remove noise, improve the SNR, and increase the resolution of the image to a super-resolution level simultaneously. This investigation shows a new and effective approach to improving image resolution and SNR. It can be applied to wide-field and confocal microscopes, which are restricted by the optical diffraction limit, as well as to super-resolution microscopes, to further improve their imaging performance.

## Figures and Tables

**Table 1:** PSNR and SSIM of the ground truth images applied with Gaussian noise and after DWDC processing.

Gaussian Noise		PSNR before Processing (dB)	PSNR after Processing (dB)	SSIM before Processing	SSIM after Processing
Average	STD				
0	2	19.5773	20.6765	0.0887	0.7082
20	4	15.9681	19.7943	0.0425	0.4916
40	6	12.8995	18.7648	0.0330	0.3908
40	10	12.1049	17.8139	0.0297	0.3251

## Supplementary Materials

The following supporting information can be downloaded at: https://www.mdpi.com/article/10.3390/mi13060824/s1. Supplementary video S1: three-dimensional reconstruction of the microtubule skeleton of 3T3 cells (before processing); supplementary video S2: three-dimensional reconstruction of the microtubule skeleton of 3T3 cells (after processing).

## References

[1] Rajadhyaksha M., Grossman M., Esterowitz D., Webb R.H., Anderson R.R. (1995). In Vivo Confocal Scanning Laser Microscopy of Human Skin: Melanin Provides Strong Contrast. J. Investig. Dermatol..

[2] Hein B., Willig K.I., Hell S.W. (2008). Stimulated emission depletion (STED) nanoscopy of a fluorescent protein-labeled organelle inside a living cell. Proc. Natl. Acad. Sci. USA.

[3] Gustafsson M.G.L. (2000). Surpassing the lateral resolution limit by a factor of two using structured illumination microscopy. J. Microsc..

[4] Zanella R., Zanghirati G., Cavicchioli R., Zanni L., Boccacci P., Bertero M., Vicidomini G. (2013). Towards real-time image deconvolution: Application to confocal and STED microscopy. Sci. Rep..

[5] Pan S., Yan K., Lan H., Badal J., Qin Z. (2020). Adaptive step-size fast iterative shrinkage-thresholding algorithm and sparse-spike deconvolution. Comput. Geosci..

[6] Sage D., Donati L., Soulez F., Fortun D., Schmit G., Seitz A., Guiet R., Vonesch C., Unser M. (2017). DeconvolutionLab2: An Open-Source Software for Deconvolution Microscopy. Methods.

[7] Periasamy A., Skoglund P., Noakes C., Keller R. (2015). An evaluation of two-photon excitation versus confocal and digital deconvolution fluorescence microscopy imaging in Xenopus morphogenesis. Microsc. Res. Tech..

[8] Khetkeeree S. (2020). Optimization of Lucy-Richardson Algorithm Using Modified Tikhonov Regularization for Image Deblurring. J. Phys. Conf. Ser..

[9] Zhao L.N., Hu W.B., Cui L.H. (2012). Face Recognition Feature Comparison Based SVD and FFT. J. Signal Inf. Process..

[10] Cameron D.G., Moffatt D.J., Mantsch H.H., Kauppinen J.K. (1981). Fourier Self-Deconvolution: A Method for Resolving Intrinsically Overlapped Bands. Appl. Spectrosc..

[11] Ali M.N. (2019). A wavelet-based method for MRI liver image denoising. Biomed. Eng. Biomed. Tech..

[12] Ali M., Chang W.A. (2015). Comments on “Optimized gray-scale image watermarking using DWT-SVD and Firefly Algorithm”. Expert Syst. Appl..

[13] Dumic E. (2010). New image-quality measure based on wavelets. J. Electron. Imaging.

[14] Dumic E., Grgic S., Grgic M. (2014). IQM2: New image quality measure based on steerable pyramid wavelet transform and structural similarity index. Signal Image Video Process..

[15] Abdulrahman A.A., Rasheed M., Shihab S. (2021). The Analytic of Image Processing Smoothing Spaces Using Wavelet. J. Phys. Conf. Ser..

[16] Liby J.J., Jaya T. (2022). Data Hiding Scheme for Video Watermarking Using Horizontal and Vertical Coefficients of Single Level Discrete Wavelet Transform Method. J. Circuits Syst. Comput..

[17] Wang H., Rivenson Y., Jin Y., Wei Z., Ozcan A. Cross-Modality Deep Learning Achieves Super-Resolution in Fluorescence Microscopy. Proceedings of the CLEO: Science and Innovations.

[18] Shajkofci A., Liebling M. (2020). Spatially-Variant CNN-Based Point Spread Function Estimation for Blind Deconvolution and Depth Estimation in Optical Microscopy. IEEE Trans. Image Process..

[19] Qin P., Cai Y., Wang X. (2021). Small Waterbody Extraction With Improved U-Net Using Zhuhai-1 Hyperspectral Remote Sensing Images. IEEE Geosci. Remote Sens. Lett..

[20] Schaap I.A.T., Carrasco C., de Pablo P.J., MacKintosh F.C., Schmidt C.F. (2006). Elastic response, buckling, and instability of microtubules under radial indentation. Biophys. J..

[21] Nagorni M., Hell S.W. (1998). 4Pi-Confocal Microscopy Provides Three-Dimensional Images of the Microtubule Network with 100- to 150-nm Resolution. J. Struct. Biol..

[22] Ogier A., Dorval T., Genovesio A. Inhomogeneous deconvolution in a biological images context. Proceedings of the 2008 IEEE International Symposium on Biomedical Imaging: From Nano to Macro.

[23] Li J., Luisier F., Blu T. (2017). PURE-LET image deconvolution. IEEE Trans. Image Process..

[24] Carlavan M., Blanc-Feraud L. (2012). Sparse Poisson Noisy Image Deblurring. IEEE Trans. Image Process..

[25] Liu H., Zhang Z., Liu S., Liu T., Yan L., Zhang T. (2015). Richardson–Lucy blind deconvolution of spectroscopic data with wavelet regularization. Appl. Opt..

[26] Sroubek F., Milanfar P. (2012). Robust Multichannel Blind Deconvolution via Fast Alternating Minimization. IEEE Trans. Image Process..

[27] Javaran T.A., Hassanpour H., Abolghasemi V. (2017). Non-blind image deconvolution using a regularization based on re-blurring process. Comput. Vis. Image Underst..

[28] Gong D., Tan M., Shi Q., van den Hengel A., Zhang Y. (2019). MPTV: Matching Pursuit-Based Total Variation Minimization for Image Deconvolution. IEEE Trans. Image Process.

[29] Willig K.I., Kellner R.R., Medda R., Hein B., Jakobs S., Hell S.W. (2006). Nanoscale resolution in GFP-based microscopy. Nat. Methods.

[30] Hell S.W. (2007). Far-Field Optical Nanoscopy. Science.

[31] Bioucas-Dias J.M. (2006). Bayesian wavelet-based image deconvolution: A GEM algorithm exploiting a class of heavy-tailed priors. IEEE Trans Image Process..

[32] Vonesch C., Unser M. (2008). A fast thresholded landweber algorithm for wavelet-regularized multidimensional deconvolution. IEEE Trans Image Process..

[33] Figueiredo M.A., Nowak R.D. (2003). An EM algorithm for wavelet-based image restoration. IEEE Trans. Image Process..

[34] Figueiredo M.A.T., Nowak R.D. A bound optimization approach to wavelet-based image deconvolution. Proceedings of the IEEE International Conference on Image Processing.

[35] Peng Y., Li Q. (2014). Wavelet transform-based feature extraction for ultrasonic flaw signal classification. Neural Comput. Appl..

[36] Tam N.W.P., Lee J.-S., Hu C.-M., Liu R.-S., Chen J.-C. (2011). A Haar-wavelet-based Lucy–Richardson algorithm for positron emission tomography image restoration. Nucl. Instrum. M ethods Phys. Res. Sect. A Accel. Spectrometers Detect. Assoc. Equip..

[37] Lussana C., Nipen T.N., Seierstad I.A., Elo C.A. (2021). Ensemble-based statistical interpolation with Gaussian anamorphosis for the spatial analysis of precipitation. Nonlinear Process. Geophys..

[38] Platte R.B., Driscoll T.A. (2006). Polynomials and Potential Theory for Gaussian Radial Basis Function Interpolation. Siam J. Numer. Anal..

[39] Hell S.W. (2002). Increasing the Resolution of Far-Field Fluorescence Light Microscopy by Point-Spread-Function Engineering. Top. Fluoresc. Spectrosc..

[40] Hell S.W., Wichmann J. (1994). Breaking the diffraction resolution limit by stimulated emission: Stimulated-emission-depletion fluorescence microscopy. Opt. Lett..

[41] Pustelnik N., Benazza-Benhayia A., Zheng Y., Pesquet J.-C. (2017). Wavelet-Based Image Deconvolution and Reconstruction. Wiley Encyclopedia of Electrical and Electronics Engineering.

[42] Richardson W.H. (1972). Bayesian-Based Iterative Method of Image Restoration. J. Opt. Soc. Am..

[43] Sfakianakis N., Peurichard D., Brunk A., Schmeiser C. (2018). Modelling cell-cell collision and adhesion with the filament based lamellipodium model. BioMath.

[44] Bonforti A., Duran-Nebreda S., MontañEz R., Solé R. (2016). Spatial self-organization in hybrid models of multicellular adhesion. Chaos.

